# How to value safety in economic evaluations in health care? A review of applications in different sectors

**DOI:** 10.1007/s10198-019-01076-9

**Published:** 2019-06-06

**Authors:** Meg Perry-Duxbury, Job van Exel, Werner Brouwer

**Affiliations:** 1 Erasmus School of Health Policy & Management, Erasmus University Rotterdam, P.O. Box 1738, 3000 DR Rotterdam, The Netherlands; 2 Erasmus School of Economics, Erasmus University Rotterdam, P.O. Box 1738, 3000 DR Rotterdam, The Netherlands

**Keywords:** Literature review, Stated preferences, Public health, Safety, I10, I12, I18

## Abstract

Improving (feelings of) safety is an important goal of many health systems, especially in the context of recurrent threats of pandemics, and natural disasters. Measures to improve safety should be cost-effective, raising the issue of how to value safety. This is a complex task due to the intangible nature of safety. We aim to synthesize the current empirical literature on the evaluation of safety to gain insights into current methodological practices. After a thorough literature search in two databases for papers from the fields of life sciences, social sciences, physical sciences and health sciences that empirically measure the value of increasing safety, 33 papers were found and summarized. The focus of the research was to investigate the methodologies used. Attention was also paid to theoretical papers and the methodological issues they present, and the relationship between safety and three categories of covariate results: individual characteristics, individual relationship with risk, and study design. The field of research in which the most papers were found was environmental economics, followed by transportation and health. There appeared to be two main methods for valuating safety: Contingent Valuation and Discrete Choice Experiments, within which there were also differences—for example the use of open or dichotomous choice questions. Overall this paper finds that there still appears to be a long way ahead before consensus can be attained about a standardised methodology for valuating safety. Safety valuation research would benefit from learning from previous experience and the development of more standardised methods.

## Introduction

Many of today’s societies are governed by rules, regulations and protocols, many of which are designed with the aim of keeping citizens ‘safe’. Safety can be defined as ‘the condition of being protected from or unlikely to cause danger, risk or injury’ (Merriam Webster Dictionary 2016). With recurrent news about threats of global warming, terrorist attacks, pandemics and natural disasters, it is no surprise that safety is a significant concern for citizens, companies, and governments. All wish to minimize the possibility of death, damage, illness or injury. However, a question that is increasingly relevant in these same societies is whether policies that aim to increase the safety of citizens, not only in the health sector, but also in for instance the transport or environmental sector, provide good value for money. After all, public money can be spent only once and investments in increased safety displace other (worthwhile) investments. To evaluate the efficiency of these policies, safety needs to be valued. Due to safety being an intangible, non-monetary good, economists tend to value risk- or uncertainty-reductions instead of ‘safety’ [9, 33, 34], with risk-reduction being the most tangible and, therefore, the most applied definition in the literature. This being said, there is no ‘golden standard’ for safety valuation. Early approaches were based on life insurance premiums, which were then replaced, initially by human capital methods, and more recently by stated preference methods [4]. This ongoing shift in approaches shows that valuing safety is a field in which methods are frequently evolving.

Research into the topic of valuing safety is scarce, scattered across scientific fields, and no review of safety valuation literature is currently available. However, (the value of) safety is likely to become increasingly important in health (economics) and beyond. Large scale surveillance systems to prevent or mitigate the consequences of pandemics by early detection of outbreaks and early determination of their causes are an example of improving safety. Other examples with direct health consequences are improved safety by stricter regulations for food production, hospital procedures or air pollution. In evaluating such measures and policies, the value of safety may be a crucial element, but little is known as to how to best capture it.

Therefore, the aim of this paper is to present a review of the existing literature; synthesizing the methodologies used in empirical research papers that value safety. The reviewed papers come from different scientific fields, including environmental economics, transport economics, food safety, crime, and health economics—indicating that the results presented in this paper may be beneficial to any future research that requires safety valuation. As the direct outcomes from these various fields are incomparable (e.g. the value of reduced risk of flooding versus the value of reduced risk of train accidents), the focus of this study is on the methodology of valuation and the characteristics of respondents, context and study design associated with elicited values of safety, as these are the most comparable aspects of the papers. Subsequently, we will emphasize the implications for valuing safety in the context of health.

The main aim of this paper is to give a review of the methods used in *empirical* research on valuing safety. Such empirical research should be embedded in theoretical research on valuing safety, and also the interpretation of empirical studies ideally is informed by such theoretical insights. Therefore, the structure of this paper is as follows. First, Sect. 2 discusses the theoretical background to the valuation of safety. Thereafter, in Sect. 3, the methods of the literature search are discussed, followed by the findings of the research (Sect. 4). Finally, we discuss the results with a special focus on lessons for valuing safety in health.

## Theoretical background

One of the ways to compare alternative policies or interventions is by applying a (form of) cost–benefit analysis (CBA), in which the costs and benefits of the alternatives in question are compared between and within said alternatives [14]. To compare the benefits from interventions that differ in outcome—for example an improvement in road safety versus an improvement in city air quality—these benefits must be expressed in a comparable metric, traditionally often in monetary terms. Even in health care, where other outcome measures are sometimes used, such as Quality-Adjusted Life-Years to express health outcomes in cost-utility analysis, other costs and benefits are typically expressed in monetary terms.

When taking an often advocated societal perspective in the evaluation [23], all costs and benefits need to be included in the evaluation regardless of where or when they fall in society. If some of the benefits (not included in QALYs) involve non-marketed goods, these goods need to be included and hence valued. The two main approaches of assigning monetary value to non-market goods are revealed and stated preference. The revealed preference approach uses observed prices and choices to derive the value of a given outcome, while the stated preference approach elicits preferences from hypothetical choices, for instance through surveys or choice experiments, to measure how an individual values the chosen non-market good [10]. Using stated preferences is more common in valuing non-market goods, as it is hard to find real world observations from which revealed preferences and valuations can be derived univocally. The most common types of stated preference studies used to value non-market goods are contingent valuation (CV) studies and discrete choice experiments (DCE). CV studies directly ask individuals their valuation in terms of willingness to pay (WTP) for some non-market good, given a certain hypothetical scenario [44], whereas DCEs also use a hypothetical scenario, but ask respondents to choose between options with several different attributes to indirectly extract their valuation [49].

In any valuation, three aspects are crucial: (1) what is being valued, (2) how it is being valued and (3) who is valuing the good on offer. These three aspects are briefly addressed below.

In terms of *what* is being valued, in the instance of safety valuation, ‘safety’ is very complex to define and, therefore, it can be easier to think of an improvement of safety being a reduction of risk of some adverse event occurring, a reduction of uncertainty, or the reduction of the impact of a specific incident which is perceived to be unsafe. However, even with a more tangible definition of safety, several issues still arise when trying to valuate it. A first issue relates to safety itself and it is that being protected has an objective and a subjective element. An example of the difference can be found in situation where objective crime figures are going down, but subjective feelings of safety do not improve. From a utilitarian perspective, one may claim that there can be value in both improving objective safety (fewer victims, less damage) and subjective safety (a stronger feeling of safety may lead to higher utility). Therefore, improving only subjective but not objective safety may still produce benefits and value. Most empirical studies deal with valuing ‘objective risks’, but it needs noting that what exactly is being valued matters.

This is also true for the type of ‘event’ that individuals are kept safe from. Of course, one would expect, ceteris paribus, improved safety from death to be valued higher than improved safety from a mild illness. In some cases, these differences may be less obvious and differ between respondents. For example, individuals may ‘dread’ certain situations more than they dread others. To illustrate this with the example of avoiding deaths, people may fear certain types of death more than others. For instance, they may fear immediate deaths more than a ‘more gradual’ process of dying. Similarly, people may be more willing to pay for safety from ‘bad deaths’, such as murder and drowning [16], than from other types of deaths. This is relevant to consider in interpreting (the heterogeneity of) results. Whether or not such differences affect final results of an economic evaluation also depends on aspects such as baseline risks [16], but for the valuation exercise these differences emphasise the importance of being clear about what is being valued.

Similarly, and relevant in the context of safety in health and other domains, is the concept of a *catastrophe.* Some safety measures are aimed at prevented large scale impacts, such as pandemics of deathly diseases or floods of large areas of some country or region. Such contexts of a valuation exercise may invoke responses reflecting that ‘large concentrated losses are over-counted relative to dispersed losses’ [56]—for example a plane crash in comparison to a number of car accidents leading to similar health losses. In a catastrophe, when risk reduction is only described in terms of a reduction in victims, this may undervalue the impact on the feeling of safety in other people. Such contexts show the interconnectedness of objective and subjective safety and it is important to understand and, if possible, distinguish these in the context of valuing safety. Especially catastrophes may have far-reaching spill-over effects and, therefore, studies valuing reduction in risk of an outcome that may be perceived as a catastrophe may need to include additional information or measures [56].

In terms of *how* safety is being valued some remarks also need to be made, next to the general observations about stated and revealed preference as well as contingent valuation mentioned above. When developing any valuation measure it is important to consider the impact that the design of the study could have on the results. One design feature that has been found to be relevant in safety valuation, related to the issues discussed above, is the information provided in the survey. Having a clear and comprehensive valuation exercise is important especially when using indirect methods, as respondents can easily be overloaded with respondent fatigue. Including too much or too little information about what is being valued could make questions harder for respondents to understand or lead to own interpretations of the question posed. How to present the information is also an important consideration. It can be presented using various survey techniques. For example, Mattea et al. [9] explore the use of visual information in a stated preference study and find that respondents’ preferences exhibited more stability when visual information was used to explain risk probabilities when studying risk reduction valuation in landslide programmes.

In CV studies ordering effects, embedding effects and internal consistency have been shown to be important [31]. Ordering effects refer to the fact that the way in which a respondent values a certain good is dependent on the order of the information presented to them during the valuation exercise [38]. Embedding effects are most relevant when referring to the valuation of public goods or services, for example a flu-vaccination campaign. By asking an individual their WTP for this campaign, they are implicitly being asked their WTP for an injection, a reduction in the probability of getting the flu, an increase in the probability of side-effects from a vaccine, etc. There are multiple ‘products’ *embedded* in this one question [38]. Internal consistency is not frequently tested in CV research, which has worried critics. In the case of CV, internal consistency refers to the fact that the same type of survey on different WTP questions should come up with consistent results. Halvorsen [31] researched ordering effects and internal consistency when testing WTP for reduced health damage from air pollution and found considerable and significant ordering effects, but could not reject their hypothesis of internal consistency. Halvorsen [31] did not specifically research embedding effects, but emphasised the complications of combining all the elements of a certain programme into one valuation question.

In terms of *who* is valuing safety, it needs noting that individual characteristics can affect the valuation. The most frequently researched of these individual characteristics is *risk perception.* This refers to how an individual perceives the level of risk in a situation [50]. High risk-perception (i.e. assuming larger levels of risk than objectively present) has been shown to lead people to value safety (or risk reduction) more highly [30]. An issue related to risk perception is probability weighting, a part of general prospect theory. Individuals are known to not evaluate probabilities linearly but to overestimate small probabilities and underestimate large probabilities [39]. In fact, Bleichrodt and Eeckhoudt [7] showed that correcting for probability weighting strongly affects the WTP estimates for reductions in health risks. Another individual issue to consider is respondent uncertainty. It has been shown that respondents are frequently uncertain about their preferences when answering contingent valuation questions and it is a concern that this uncertainty may be affecting CV results [41]. However, Logar and van den Bergh [41] found that incorporating information on respondent uncertainty into the model does not lead to any gains compared to a standard CV model. It is also worth noting that risk perception is rarely equivalent to worry, as worry is based on emotion rather than intellectual judgment. As Sjoberg [50] puts it: ‘One can feel worried about a risk without believing that it is especially large, and *vice versa’*. However, worry and also pessimism have been shown to be small explanatory factors of risk perception that vary in size depending on the risk being studied [50].

Another issue that is frequently thought of as causing bias in CV results is public opinion. Critics have contested the assumption underlying CV that respondents have ‘well-defined and self-interested preferences’ and argue that respondents are in fact influenced by public opinion. Chanel et al. [15] attempted to test this by giving a group of respondents the option to revise their answers on how much they were willing to pay for a decrease in air pollution after hearing the mean WTP response from the survey group they were in [15]. They found that at least this type of ‘public opinion’ had no significant impact on respondents’ answers and suggest that it may be a poorly defined private value structure (or preferences) that leads to a reaction to public opinion [15]. The fact that (ideas about) public opinion may have an impact on valuations of safety at least may be something that those developing a CV study may wish to bear in mind.

From the above it is clear that valuations of safety may depend on the context provided in describing what is being valued, on how safety is valued and by whom. So far, a golden standard for performing valuation studies of safety emerging from theory is lacking. Hence, it is important to consider how safety is valued in practice.

## Methods

In October, 2016, a comprehensive literature search for papers related to the valuation of safety was performed. We assumed that alongside papers related to health, there would also be interesting methods on the valuation of safety outside of the biomedical fields. Therefore, one biomedical database, Embase, and one ‘broader’ database, Scopus, were used for the search. Embase was chosen as the biomedical database as it holds the largest number of indexed records (in comparison to PubMed and Medline), and also includes all records that are present in Medline. Practically, Embase has a somewhat more advanced search filter than other biomedical databases. Scopus was chosen as it covers a broad range of subject fields: life sciences, social sciences, physical sciences and health sciences, and it is comparable to Web of Science.

There was no restriction on time period. Book chapters, dissertations, and theses were not considered. The following terms were used for the search: value, valuation, review, shadow price, willingness to pay, willingness to accept, discrete choice experiment, stated preference, revealed preference, and contingent valuation. The above terms were used in combination with these search terms: Safety, security, uncertainty reduction, risk reduction. The exact search strings are provided in Appendix A. Secondary references were found by searching the references of the already included papers to find relevant papers that the databases may not have included.

Papers retrieved from the search were selected for review if they fitted both of the following inclusion criteria: First, the research is empirical, and second, the research deals with the valuation of safety, security, risk reduction, uncertainty reduction or reduction of some event that is stated to decrease safety. Papers were excluded if safety valuation was not a main objective of the paper, or if the paper was not in English (Table 1).

**Table 1:** Results of Search Terms

	Safety	Security	Uncertainty reduction	Risk reduction	Total
Embase					
Value	29,099	2409	15	3312	34,835
Valuation	173	61	1	84	319
Shadow price	1	2	0	0	3
Review	177,856	9016	15	24,150	211,037
WTP	252	24	0	141	417
WTA	41	4	0	8	53
DCE	61	1	0	25	87
Stated preference	32	1	0	21	54
Revealed preference	2	0	0	3	5
CV	10	5	0	10	25
Total (incl. Value and Review)					246,835
Total (excl. Value and review)					963
Scopus					
Value	82,152	30,435	4535	25,783	142,905
Valuation	706	1218	143	531	2598
Shadow price	11	41	4	8	64
Review	194,236	20,204	1990	67,514	283,944
WTP	632	181	97	497	1407
WTA	135	58	5	59	257
DCE	93	16	4	70	183
Stated preference	274	82	13	138	507
Revealed preference	310	128	8	101	547
CV	85	37	11	87	220
Total (incl. Value & review)					432,632
Total (excl. Value & review)					5783

One of the authors (MP) screened the title and abstract of each paper, checking for inclusion and exclusion criteria. After this screening a second check was performed in which entire texts were scanned to ensure the papers were eligible for the review. The following information was extracted and entered into a table (Table 2) for all included papers:

**Table 2:** General Paper Information

Author(s)	Title of study	Year	Academic field	Definition of safety	Elicitation format
Alberini et al. [1]	Willingness to pay to reduce mortality risks: evidence from a three-country contingent valuation study	2006	Health	Risk reduction	Contingent valuation
Andersson [2]	Willingness to pay for road safety and estimates of the risk of death: evidence from a Swedish contingent valuation study	2012	Transport	Risk reduction	Contingent valuation
Atkinson et al. [3]	Valuing the costs of violent crime: a stated preference approach	2015	Crime	Incidence reduction	Contingent valuation
Carlsson et al. [12]	Is transport safety more valuable in the air?	2004	Transport	Risk reduction	Contingent valuation
Carlsson and Johansson-Stenman [11]	Willingness to pay for improved air quality in Sweden	2000	Environment	Incidence reduction	Contingent valuation
Carson and Mitchell [13]	The value of clean water: the public’s willingness to pay for boatable, fishable, and swimmable quality water	1993	Environment	Incidence reduction	Contingent valuation
Chanel et al. [15]	Does public opinion influence willingness-to-pay? Evidence from the field	2006	Environment	Risk reduction	Contingent valuation
Corso et al. [17]	A Comparison of willingness to pay to prevent child maltreatment deaths in Ecuador and the United States	2013	Health	Incidence reduction	Contingent valuation
Dealy et al. [19]	The economic impact of project MARS (Motivating Adolescents to Reduce Sexual Risk)	2013	Health	Risk reduction	Contingent valuation
Determann et al. [20]	Acceptance of vaccinations in pandemic outbreaks: a discrete choice experiment	2014	Health	Incidence reduction	Discrete choice experiment
Dickinson and Paskewitz [20]	Willingness to pay for mosquito control: How important is west nile virus risk compared to the nuisance of mosquitoes?	2012	Environment	Incidence reduction	Conjoint analysis
Enneking [24]	Willingness-to-pay for safety improvements in the German meat sector: the case of the Q&S label	2004	Food safety	Safety	Discrete choice experiment
Flügel et al. [25]	Car drivers’ valuation of landslide risk reductions	2015	Environment	Risk reduction	Discrete choice experiment
Garza-Gil et al. [26]	Marine aquaculture and environment quality as perceived by Spanish consumers. the case of shellfish demand	2016	Environment	Safety	Contingent valuation
Georgiou et al. [27]	Determinants of individuals’ willingness to pay for perceived reductions in environmental health risks: a case study of bathing water quality	1998	Environment	Risk reduction	Contingent valuation
Gerking, et al. [28]	The marginal value of job safety: a contingent valuation study	1998	Labour	Risk reduction	Contingent valuation
Gyrd-Hanssen et al. [29]	Willingness-to-pay for a statistical life in the times of a pandemic	2007	Health	Risk reduction	Contingent valuation
Haddak et al. [30]	Willingness-to-pay for road safety improvement	2014	Transport	Risk reduction	Contingent valuation
Halvorsen [31]	Ordering effects in contingent valuation surveys: willingness to pay for reduces health damage from air pollution	1996	Environment	Risk reduction	Contingent valuation
Henson [33]	Consumer willingness to pay for reductions in the risk of food poisoning in the UK	1996	Food safety	Risk reduction	Contingent valuation
Hunter et al. [36]	The effect of risk perception on public preferences and willingness to pay for reductions in the health risks posed by toxic cyanobacterial blooms	2012	Environment	Risk reduction	Contingent valuation
Iraguen and de Dios Orutzar [37]	Willingness-to-pay for reducing fatal accident risk in urban areas: an internet-based web page stated preference survey	2004	Crime	Risk reduction	Discrete choice experiment
Khan et al. [40]	Household’s willingness to pay for arsenic safe drinking water in Bangladesh	2014	Environment/health	Risk reduction	Contingent valuation
Loureiro and Umberger [42]	A choice experiment model for beef: What US consumer responses tell us about relative preferences for food safety, country-of-origin labeling and traceability	2007	Food Safety	Safety	Discrete choice experiment
Mattea et al. [43]	Valuing landslide risk reduction programs in the Italian Alps: The effect of visual information on preference stability	2016	Environment	Risk reduction	Discrete choice experiment
Mofadal et al. [44]	Analysis of pedestrian accident costs in Sudan using the willingness-to-pay method	2015	Transport	Risk reduction	Contingent valuation
Patil et al. [46]	Public preference for data privacy—a pan-European study on metro/train surveillance	2016	Transport	Security	Discrete choice experiment
Pham et al. [47]	Households’ willingness to pay for a motorcycle helmet in Hanoi, Vietnam	2008	Transport	Incidence reduction	Contingent valuation
Rizzi and Ortuzar [48]	Stated preference in the valuation of interurban road safety	2003	Transport	Safety	Discrete choice experiment
Smith et al. [51]	How should the health benefits of food safety programs be measured?	2014	Food safety	Risk reduction	Discrete choice experiment
Viscusi [52]	Valuing risks of death from terrorism and natural disasters	2009	Environment	Risk reduction	Discrete choice experiment
Yabe [54]	Students, faculty, and staff’s willingness to pay for emergency texting	2016	Crime	Safety	Contingent valuation
Yun et al. [55]	Analysis of the relationship between risk perception and willingness to pay for nuclear power plant risk reduction	2016	Environment	Risk reduction	Contingent valuation

1. Author(s)
2. Title of Paper
3. Year
4. Academic Field
5. Definition of safety
6. Method

Two separate tables (Tables 3 and  4) were made for each type of method with columns for:

**Table 3:** Contingent Valuation Method

Paper	Scenario description	CV question(s) asked to respondents	Measurement scale	Econometric model(s)	Covariate results
Crime					
Atkinson et al.	Respondents shown injury descriptions for 3 types of assault: common assault, other wounding, serious wounding. Also informed of pre-policy risk of the incident occurring	Asked WTP to reduce chance of being a victim to one of the three offences (randomized per respondent) by 50% over the next 12 months. Payment vehicle is a one-off increase in local changes for law enforcement	Payment card: £0-5000	Interval data model	Severity of the risk increases WTP. Higher incomes and education both increase WTP
Corso et al.	Respondents are asked to imagine that there is a program available in their city that reduces the risk of a child being killed by a parent or caretaker by 50%	Asked WTP for this program through (1) taxes or (2) donations	Double-bounded dichotomous choice: Initial WTP value between $10 and $300. Second WTP values are $25 higher (lower) if response is ‘yes’ (‘no’)	Maximum likelihood function	Those reporting history of child maltreatment have lower WTP
Yabe	Respondents are told that a text-to-911 service would be paid for by a few charges to students, staff and faculty at the university	Respondents are asked if they would be willing to pay X$ for an emergency text messaging service	Dichotomous choice: bids—$1, $2, $3, $5, $10	Logit model	Being interested in emergency texting, having experience in campus emergencies, being older, having a higher income, and being American (rather than international) leads to higher WTP
Environment					
Carlsson and Johansson-Stenman	No scenario given, researchers want respondents to judge the information about air pollution from various sources	Asked WTP for a 50% reduction in concentration of harmful substances where they live and work	Open-ended questions	Probit. Tobit type I. Tobit type II. Independent models	WTP increases in income, wealth and education. WTP is larger for: men, members of environmental organizations, people living in big cities, and those who own their house or apartment. WTP is lower for retired people
Carson and Mitchell	Respondents told that although present minimal water level is ‘boatable’, most of the nation’s freshwater bodies are fishable and 70% are swimmable. Used the ‘Resources for the Future’ water quality index to show physical water quality parameters	Asked WTP in taxes ‘to keep the nation’s freshwater bodies from falling below the boatable/fishable/swimmable level where they are now’. Four WTP amounts solicited for each of the three water quality questions: (1) amount given for each of the WTP questions (2) WTP given after first amount is repeated and respondents encouraged to make desired corrections (3) WTP after respondents informed of the range of the amounts households in their income group were already paying for water quality (4) WTP given after respondents pushed to increase their bid	Payment card: 0$ to a ‘very high amount’. Five points on the card show average amounts households pay in taxes for non-environmental public goods	Cobb–Douglas	N/A
Chanel et al.	Respondents are given the hypothetical choice to move with their family to a less polluted city. Two cities are proposed that are equivalent with the exception of level of pollution and the cost of living	Four steps: (1) WTP to live in less polluted city. (2) WTP after shown mean WTP of all respondents. (3) WTP after receiving scientific and quantitative information on health effects of pollution. (4) WTP after new mean shown to respondents	Dichotomous choice questions. Open-ended questions	Wilcoxon sign-ranked tests	Public opinion has no effect on WTP. Information provided leads to higher WTP
Garza-Gil et al.	No scenario provided	Asked WTP for an enhanced safety guarantee programme for shellfish quality and environmental conditions	Dichotomous choice questions. WTP 5%, 10%, 20% or more than 20% more than normal price	N/A	The higher the price the lower the number of people WTP for the intervention
Georgiou et al.	Respondents informed about sewage contamination of bathing water and health risks from bathing, EC bathing water standards and actual quality of water at a particular beach	Asked WTP for (1) for a gain (2) for a loss in bathing water standards—dependent on the beach at which applicants are surveyed	Open questions	Semilog model	Higher income and education lead to a higher WTP. The more unacceptable the respondent finds the risk, the higher their WTP. Having a family member who has suffered due to poor bathing water leads to a higher WTP
Halvorsen	Respondents given description of benefits from a 50% reduction in air pollution. These are (1) reduction in in the risk of becoming ill and (2) a reduction in damage due to acid rain	Asked maximum WTP for 50% reduction in air-pollution. Four sub-samples with two splits: (1) Sub-samples B and D are told that the government will subsidize electric cars. A and C are told that the government uses a package of unspecified tools. (2) Those in A and B are given all information, then asked WTP. Those in C and D are first given health effect information then asked WTP, then are given all other effects and asked if they wish to change their WTP	Open questions	Tobit. Cragg (i) Probit model (ii) Truncated model	Income, living in a major city, having a university degree and being concerned with the environment all have a positive effect on WTP. Age has a negative effect on WTP
Hunter et al.	Respondents informed about (1) what cyanobacteria are (2) the ecological and human health problems they cause and (3) the practical options available for health-risk mitigation at Loch Leven	Asked max WTP towards measures to reduce number of Risk Days from 90 to (1) 45 or (2) 0. Payment vehicle is the cost of domestic water supply set by the council	Payment card (values not stated)	Binary logit model. Non-parametric models: normal, logistic, lognormal, Weibull, and spike model	Those with higher concern for environmental health risks have higher WTP. Income has a positive effect on WTP
Khan et al.	No scenario provided	Asked WTP for: (1) a communal deep tube well, (2) one-time-off capital investment costs of the well (3) one-time-off investment costs and (4) operation and maintenance costs	Double bounded dichotomous choice. Capital costs: Min. bid—50 BDT. Max. Bid—250 BDT. O&M costs: min. bid—10 BDT. Max. Bid—100 BDT	Bivariate probit model. random effects probit	When respondents are male or earn higher incomes WTP is higher. If households are exposed to higher risk levels, if respondents are aware that their water is contaminated, and if household members are affected by arsenic exposure then WTP increases
Yun et al.	Respondents are first asked to rank an image about nuclear power plants on a Likert scale of ‘very good image/safe (5)’ to ‘very bad image/unsafe (1)’	Respondents are asked if they would pay *A$* to reduce NPP hazard	Dichotomous choice. Bids are not described	Log-linear. Linear. Linear-log. Power regression models	Higher scientific background/low risk perception led to a lower mean WTP. Mean WTP decreased with increasing quality of informational image
Food safety					
Henson	Respondents are informed that chicken/egg consumption can cause food poisoning. They are told about two brands of chicken/eggs in the shop. Brand A and Brand B are identical except Brand A has been thoroughly tested and thus has a lower risk of giving one food poisoning	Maximum additional amount WTP for a risk reduction in (1) fatal food poisoning (2) mild food poisoning (3) moderate food poisoning (4) severe food poisoning in chicken or eggs	Open question	Ratios calculated	More severe outcome leads to higher WTP. Personal experience of food poisoning has a negative effect on WTP. Mean WTP is higher for female respondents. Age and education both have a negative effect on WTP. WTP is positively affected by income
Health					
Alberini et al.	Respondents are shown their baseline risk of death (that varies with gender and age) over the next 10 years	Asked WTP for a risk-reduction in death of (1) 5-in-1000 incurred over the next 10 years, (2) 1-in1000 incurred over the next 10 years, (3) 5-in-1000 that begins at age 70 and is spread over next 10 years. Payment would be made every year	Dichotomous choice questions	Accelerated-life Weibull model	Income increases WTP. WTP increase with age until age 60 and then plateaus. Hospitalization for cardiovascular or respiratory illness leads to higher WTP
Dealy et al.	Participants are randomly assigned to receive one of three treatments: (1) sexual risk reduction intervention (2) sexual risk reduction plus alcohol risk reduction (3) sexual risk reduction intervention including both an alcohol and a marijuana risk reduction component	Asked WTP ‘not to get’ (1) a curable STD (2) an incurable non-fatal STD (3) a fatal STD. Asked before and after intervention	Open question with a bound of $0-100,000	Anova	WTP increases after receiving the intervention. WTP increases with the severity of the STD
Gyrd-Hanssen et al.	No scenario provided	Asked maximum WTP to have a course of Tamiflu drug available in case they would need it	Open questions	Linear regression analysis	Age and being female increase WTP. Household income has a positive impact on WTP. Being uncertain of baseline risk has a positive impact on WTP. Being uncertain of the perceived benefit has a negative impact on WTP
Labour					
Gerking, et al.	Respondents are asked what their current job is	Asked (1) how large an increase in annual wages would lead to respondent voluntarily working ‘one step up’ the risk ladder (WTA) (2) how large a decrease in annual wages would a respondent forego to move one step lower on the risk ladder	Payment card: $0 to $6000	Two-limit tobit procedure	Higher income and perceived likelihood of death at work leads to higher WTP/WTA. Older-workers have a higher WTP/WTA. WTP decreases with formal educational levels
Transport					
Andersson	Respondents shown overall death risk for an individual. Also shown risk of dying in a traffic accident	Asked one of two questions: (1) WTP for reducing personal annual risk of death by a third. (2) WTP for reducing personal annual risk of dying in a traffic accident by one-third	Open-ended questions	Non-linear models. Log-linear models	WTP increases as baseline risk increases. WTP declines with age. WTP declines with background risk. WTP increases with income
Carlsson et al.	Two scenarios: (1) The respondent is going to take a taxi alone. They have two taxi options which are identical except for the risk of a fatal accident—1 in 1 million (AAA) or 0.5 in 1 million (BBB). (2) The respondent will take a plane alone. They have two airline options which are identical except for the risk of a fatal accident—1 in 1 million (AAA) or 0.5 in 1 million (BBB)	Cases: Asked WTP for safer air trip compared to AAA at (1) 500 SEK (2) 3000 SEK. Asked WTP for safer taxi ride compared to AAA at (3) 50 SEK (4) 500 SEK. (5) Asked both (1) and (4). (6) Asked both (2) and (4)	Open-ended questions anchored with baseline-risk prices (AAA)	Tobit type II	Cost of trip leads to a higher WTP. Higher income leads to higher WTP. Male respondents have lower WTP. Fear of flying leads to a higher WTP for both air and taxi questions
Haddak et al.	Three projects presented to respondents: reduces risk of being a victim of (1) a road accident that causes minor injuries (2) a road accident resulting in serious injuries (3) a road accident that results in moderate injuries	Asked how much they would be willing to pay for a (1) 25% reduction (2) 50% reduction in risk of experiencing various non-fatal types of injuries following a road accident	Open questions	Logit. Tobit	WTP is higher for more severe injuries. WTP increases with income. Accidental experience of individuals (direct and indirect) leads to increased WTP
Mofadal et al.	Respondent is told to imagine going to work or performing daily activities and during these they need to cross busy streets to reach their destination. The respondent can choose one of five options to reduce this risk	The respondent first chooses the optimum scenario regarding: crossing behavior and side walking. They are then asked their maximum WTP to reduce the risk of a fatality in that scenario. They are also asked their maximum WTP for a pedestrian safety program that reduces fatality risk by 50%	Payment card: 0 to more than 3000 SDP	Log-linear	Age positively affects WTP. Income positively affects WTP. Married respondents have a lower WTP. Males have a higher WTP. Higher education increases WTP
Pham et al.	Respondents are given the hypothetical situation that the government subsidizes the price of motorcycle helmets	Respondents are asked the maximum amount they are willing to pay for a motorcycle helmet	Open question. Dichotomous choice questions—min. 50,000 SDP, max. 150,000 SDP	Interval regression. Multi-linear regression model	Age and income have a positive effect on WTP. Those with higher education, those with jobs outside of the office and those with a better knowledge of/attitude towards helmets have a higher WTP

**Table 4:** Discrete choice experiment/conjoint analysis

Paper	Scenario description	Question asked to respondents	Attributes	Econometric model(s)	Covariate results
Crime					
Iraguen and de Dios Orutzar	Respondents are asked to imagine they are traveling to work from home. The trip takes place on a regular working day, they arrive at their destination at around 7:45 am, and they drive their own car and are responsible for all costs involved	Respondents are asked to choose between two different routes with differing attributes	Travel time. Travel cost. Number of fatal car accidents per year	Multinomial logit model	Income negatively affects the perception of the importance of travel cost. Safety valuation is positively affected if the individual travels with somebody else. This is also true if the respondent is female or they have been in a serious accident before
Environment					
Dickinson and Paskewitz	Respondents are informed of the multiple types of mosquitoes in Madison (some a nuisance, some transmit West Nile virus). Control program which would control mosquito larvae could control one type of mosquito larvae or both	Asked to choose between pairs of hypothetical control programmes	West Nile Risk. Type of mosquito targeted. Cost (through taxes): $10–200	Conditional logit model	Increased risk level leads to higher WTP. WTP decreases as cost increases
Flügel et al.	Respondents who had a recent trip by car were presented with different choices for a car trip route	Respondents are asked to choose between two routes with four differing attributes, 6 times	Cost: fuel and toll. Travel Time. Casualties: fatalities and serious injuries. Landslides: share of the route with landslide risk	Mixed logit models	Men are less likely to choose lower landslide risk. People with a higher education tend to choose the option with the lowest risk more often
Mattea et al.	No scenario given	Respondents are given six choice sets of seven alternatives, each of which consists of five attributes. These questions are asked twice, and respondents are given visual information on the possible options before being asked a second time	Four alternatives represent devices to protect against landslides: diverging channel, retaining basin, Video cameras, and Acoustic sensors. The fifth alternative is a hypothetical road toll	Mixed logit in WTP spaces. Multinomial logit model. Mixed logit in preference space	The ‘status quo’ is negatively perceived
Viscusi	Traffic—On an average day 100 people die due to traffic accidents. These risks are isolated deaths. Natural disasters—these are national catastrophes and large numbers of people can die at the same time. Hurricane Katrina killed over 1000 people. Terrorism—attacks by terrorists can also be catastrophes. The 9/11 attacks killed 2976 people	Respondents are asked ‘risk–risk’ tradeoff questions (traffic accident-terrorist attack, traffic accident-natural disaster) in two sets of 6 question blocks	Type of deaths prevented. Average number of deaths prevented	Conditional logit models. Mixed logit models	More education raises the utility coefficient in every instance, and more so with terrorism. Income has a negative effect on utility. Seatbelt usage increases the utility of reducing all deaths
Food safety					
Enneking	Participants are given a short introduction to the quality and safety labelling system (regarding liver sausages)	Asked to name three choices from a set of 6 sausages	Brand A: national premium brand (with/without Q&S label). Brand B: national brand (with/without label). Brand C: national premium brand—reduced fat (no label). Brand D: private label—organic (no label). Brand E: national organic umbrella brand name (no label). Brand F: private label	Maximum likelihood	Those who find low prices important avoid the more expensive labelled brands
Loureiro and Umberger	No scenario given	Asked to choose between two steaks (option A and option B) with five varying attributes	Price ($/lb). Country of origin labeled. Traceability to the farm. Food safety inspected. Guaranteed tender	Multinomial conditional logit model	Increasing price of option leads to lower utility. Steaks inspected by US food inspectors carry the highest premium
Smith et al.	No scenario given	Regarding improvement of food safety respondents are asked to choose between: the ‘status quo’, ‘Hire more inspectors’ and ‘purchase medicine’. Each subject is asked 12 choice questions, where each option consists of five attributes	Annual risk of food borne illness. Average amount of time you will be sick. Extra time needed to prepare food. Cost. Annual increase in income tax	Multinomial logit models	Consumers prefer reduction ex ante risk than ex post. Those who are more willing to accept risk, are not as likely to accept risk reduction policies. Respondents prefer private control over the risk reduction
Health					
Determann et al.	Respondents are presented with some combination of two scenario variables (1) susceptibility to the disease (2) severity of the disease.	Asked to choose between Vaccine A, Vaccine B and ‘No Vaccine’ in 16 choice sets. Vaccines are comprised of different levels of 5 attributes.	Effectiveness of vaccine. Safety of the vaccine. Advice regarding the vaccine. Media coverage. Out-of-pocket costs.	Latent class model.	Females and individuals who stated they would never get vaccinated were more influenced by media and more sensitive to costs. WTP is higher for more effective vaccines, especially if the outbreak was more serious.
Transport					
Patil et al.	No scenario given	Each respondent answered five choice exercises regarding their security preferences when traveling by train or metro	Type of CCTV cameras. How long CCTV information is stored. Who can access CCTV information. Security personnel at the station. Type of security checks at the station. Time to go through security checks. Security surcharge	Multinomial logit model	All preferred CCTV over no CCTV. Preference is weaker for younger people. Females have a stronger preference for CCTV
Rizzi and Ortuzar	Survey is disguised as a survey to improve interurban route policy and road safety. Respondents are given an identical trip in which: they drive their own car, they pay for the total cost of the trip, and they have to return after 20:00	Respondents are asked to answer nine choice situations. They are asked to choose between two routes with differences in the three attributes	Travel time. Toll charge. Annual accident rate (represents “general level of safety”)	Binary logit models	Women have a higher preference for safety than men, as do older people. There is a higher preference for safety if the trip takes place at night. A person driving with others in the car is more aware of risk

7. Paper
8. Scenario Description
9. Question asked to respondents
10. Measurement scale (CV) or Attributes (DCE)
11. Econometric Model(s)
12. Covariate results

The comprehensive search yielded a total of 679,467 results. Because the search terms ‘value’ and ‘review’ produced many seemingly irrelevant results, any results using these search terms were not included in the abstract screening, leaving 6746 results for further screening. This first involved evaluating whether paper titles appeared to fit the inclusion criteria, which resulted in the exclusion of 6659 papers (99%). If the title of the paper was relevant then the abstract was checked to confirm that the paper did indeed fit the inclusion criteria. This was frequently not the case, leaving 49 papers (5%) after this screening. The reference lists of these papers were searched for additional papers empirically examining the valuation of safety. Nine additional papers were added after this step, hence, 58 papers were included in the next step of the review process. This involved a more thorough check, which showed that 24 of the 58 papers were either a non-empirical paper or did not focus on the value of safety. One additional paper was excluded as it only measured relative values of safety rather than absolute, using a ranking method. Therefore, 33 papers were finally included and summarized in the review.

The main aim of this review, as mentioned previously, was to examine the various methodologies used for valuing safety. Therefore, in both the table and the findings section of this paper, most weight will be placed on study methodology. Due to the variety of topics covered by the papers, the comparison of WTP values seemed nonsensical (since incomparable). However, to give some insight into possible results from similar studies, the covariate results that can be compared across fields are discussed in the findings.

## Findings

Table 2 shows general information about the papers extracted from the review process. Regarding the fields of the papers, the most popular field is Environment (39%), followed by Transportation (21%) and Health (15%). Twenty-two of the papers (67%) used the contingent valuation (CV) method for their valuation of safety and 11 (33%) used a form of discrete choice experiment (DCE) or conjoint analysis. Of the 33 papers, 20 (60%) used ‘risk reduction’ as the definition of safety, seven (21%) simply referred to a ‘reduction in [unwanted outcome]’, five papers (15%) used the term ‘safety’, and one paper (3%) valued ‘security’.

Table 3 synthesizes the more specific results of the papers that use CV methods. All papers used one of three types of measurement scale: open-ended questions, payment cards or dichotomous choice questions. Dichotomous choice questions can be broken down into single- or double-bounded questions, where a double-bounded question means that, after being given an initial ‘yes or no’ WTP price, as in a single-bounded question, the respondent is then given a second WTP option dependent on his first answer [32]. The most popular question format of the 22 papers is an open-ended question (48%) [11, 15, 16, 19, 27, 29, 30, 33, 47], followed by dichotomous choice [1, 15, 17, 26, 40, 47, 54, 55] (35%), and payment card [33–37]. Two of the papers use both open-ended questions and dichotomous choice [15, 47]. Of the six papers using dichotomous choice, two use double-bounded questions [17, 40].

Table 3 also includes findings concerning covariates and their effect on WTP for safety. These covariates can be categorised into three groups: individual characteristics, individual relationship with risk, and aspects of the study design. Regarding individual characteristics, the findings show that higher income was associated with a higher WTP in every case in which it was investigated [2, 3, 12, 27–31, 33, 36, 40, 45, 47, 55]. Many papers investigating this relationship (70%) report that having a higher level of education is associated with a higher WTP [1, 11, 27, 31, 45, 47], while others (30%) report the opposite result [28, 33, 55]. Age and gender are variables for which ambiguous effects were reported. Several papers (54%) find that increasing age is associated with increased WTP [1, 28, 29, 45, 54], however, others (46%) report the opposite result [2, 11, 31, 33, 55]. In papers where gender was considered sometimes men reported a higher WTP [11, 45] and sometimes women did [12, 29, 33].

Second, we can consider the group of variables that concern the individual and their relationship with the risk. For example, if an individual is more susceptible to the outcome [1], has been previously exposed [40] to the outcome, or has a family member who has experienced the situation [27], they are associated with reporting a higher WTP according to some of the papers reviewed. There are several other factors that could lead to an increased WTP. For example, if an individual is more concerned about the issue at risk [31, 36], finds the risk unacceptable [27], has a higher perceived risk [27, 28], is uncertain of the benefit or risk of the outcome [29], or is aware of [40], interested in [54], or knowledgeable about [47] the issue. Those with experience of the outcome sometimes report higher WTP (60%) [19, 30, 54] and sometimes report lower WTP (40%) [17, 33] than those who had not experienced the outcome. The studies in which WTP is lower with experience of the outcome cover the topics of child maltreatment risk reduction [17] and the risk reduction of food poisoning [33].Corso et al. [17] indicate that the finding is not what was expected, but they do not come up with a concrete explanation for the mechanism underlying the result. Henson [33] explained his result through two mechanisms: the first is that those who have recently suffered from food poisoning believe that they have a smaller chance of getting food poisoning in the future, and the second is that many suffered only mild symptoms and so may underweight the probability of having moderate to severe food poisoning symptoms [2].

Third, we can consider the group of variables related to aspects of the study design. Using a higher baseline risk [2] or severity of risk [3, 19, 33] is associated with individuals reporting a higher WTP. From the two CV studies that place a price on the intervention, one study finds that increased cost price is associated with higher WTP [12] while the other study finds the opposite result [26]. Carlsson et al. [12] give no explanation as to why a higher cost price suggests a higher WTP in their paper, however, as they research choices between taxi rides and flights it may be due to people assuming that the more expensive the journey is, the safer it is. Two studies also investigated the effects of more information on individuals’ WTP. Chanel et al. [15] found that giving more information regarding pollution levels is associated with higher WTP, whereas Yun et al. [55] found that providing people with better quality informational images is associated with lower WTP for reduced nuclear power plant hazard. Because they approach the study from the point of view that nuclear power plants are safer than assumed by some of the public, they do not explicitly discuss why better quality information is associated with lower WTP [55], however, in general better information should have no a priori effect: it simply depends on whether prior expectations were too high or too low.

As previously mentioned, the second most popular method for valuing safety is DCE or conjoint analysis. Table 4 summarizes the main traits of the papers in which DCE or conjoint analysis is used. The most obvious difference between DCE (or conjoint analysis) and CV methods is that DCE and conjoint analysis use attributes so as to *indirectly* measure the value of what is being researched. Since the papers in this review came from many different fields, it is not possible to directly compare attributes. However, there were three types of attribute which almost all DCE studies used and can be described in broad terms as: one which considers the cost price (81%) [20, 21, 24, 25, 42, 43, 46, 48, 51], one which considers the level of risk or risk reduction (72%) [20, 21, 25, 37, 42, 48, 51, 52], and one which considers the type of intervention (81%) [20, 21, 24, 25, 42, 43, 46, 48, 51, 52].

Looking at the results from the DCE papers, the effects of covariates on WTP can, once again, be split into three groups—personal characteristics, individual relationship with risk, and aspects of the study design. From Table 4 we can see that higher age [46, 48], education [25] and income [37] all increase WTP. The only personal variable that differed from the CV results is that in the DCE studies that investigated gender differences (36%), women [20, 25, 37, 48] always reported a higher WTP. Regarding the interaction of individuals and risk; experience of the event [37] is associated with higher WTP. Finally, looking at the variables which relate to the effectiveness of the method: a higher cost price was associated with lower WTP [21, 42], while a more severe outcome [3], a higher risk level [21] and a more effective treatment [20] were all associated with higher WTP.

Many of the papers in the study consider some theoretical issues that come with the methodology used. Out of the CV papers, most of those that do consider theory look at the use of visual aids to represent risk [1–3, 12, 13, 28]. Other issues considered are sample size limitations [47, 54], embedding effects [12, 27, 31], the interpretation of risk [29, 45], and interviewing effects [36]. The most commonly considered theoretical issues in the DCE papers were sample bias [21, 37], the use of visual aids [43] and behaviour comparability [42, 48].

## Discussion

This review aimed to synthesize the methodology and study design used in empirical research valuating safety. This issue is becoming more and more relevant as economic evaluations are increasingly used in the context of informing governmental policy, and as potential threats to our safety in different areas increasingly a subject of policy. As can be seen from the results section above, there are several main findings regarding the valuation of safety. First, the two main methods used are CV and DCE (or conjoint analysis), with CV being the most frequently used. Second, most studies used ‘risk reduction’ as a definition of safety when valuating it. Third, there are covariate results other than the main variable of interest that are measured across papers, all of which fell under three categories: individual characteristics, the relationship between the individual and risk, and aspects of the study design. Overall, it was the covariate results related to individual characteristics that led to the most ambiguous conclusions, while the results concerning the individual’s relationship with risk mostly ran in the same direction across papers. Finally, while most papers did *mention* at least one of the theoretical issues related to valuing safety, few attempted to *tackle* the issues they mention.

Something that is not directly discussed in the findings but is noteworthy, is that all papers use an individual perspective when valuating safety, and none consider or mention using a societal perspective. Doing this would allow the measurement of how individuals value the safety of others and not just themselves, which is clearly relevant when policies are designed to improve the safety of citizens in general, and use taxes as the payment vehicle. However, one may then encounter the issue of double-counting, where an individual not only values their utility, but also the utility of someone else [6]. Using a societal perspective in the methodological design would involve additional scenario description and questions. For example, one can include information in the scenario description about who is at risk and who benefits from the intervention, and also ask questions about the individual’s WTP if others are also paying (e.g. through raising taxes), or if the individual themselves does or does not benefit (i.e., distinguishing between social values that do or do not take self-interest into account [8, 22]).

Several further observations can be made on the basis of this literature review. First, there is the limited number of papers retrieved from the literature search. Therefore, it is difficult to make strong conclusions or recommendations from any of the results, especially those stemming from DCE experiments, of which relatively few were included. To comment on similarities in methodologies used within fields would require a higher number of papers per field as well. Second, there is the complexity to defining safety. Even though most papers define safety as ‘risk reduction’ when valuing it, not all do, and so this muddles any comparison between papers that use different definitions. In addition, acknowledging that feelings of safety may be important for people’s wellbeing next to objectively improved safety, it should be noted that the valuations of feelings of safety were not present in the current review. Of course, improved objective risk reduction may result in feeling more safe as well, but the two need not coincide. Moreover, we may have excluded risk reduction papers that do not allude to safety, even if methodologically very similar to papers included in this review. Finally, there is the wide range of fields used in this research. Although the diversity of topics does show that the valuation of safety is relevant in many different areas, it is limits the comparison of results.

The above observations show us how useful the (evidence based) standardisation of some elements of safety valuing methodology would be. Governments are presented many policy options while they have a restricted budget. Consequently, they must make choices about which policies to implement and which not, potentially concerning different departments, such as health and education. When making such choices, information about the value for money different policies generate is relevant information and in this context a somewhat standardised methodology for valuing safety would be beneficial for the comparability of information between policies. For example it could be beneficial to have a standardised number and order of questions or attributes and levels, to require the assessment of individual risk perception and to control for probability weighting, just to name a few options.

As with any study, there are of course limitations: First, our search was purposely somewhat targeted and restrictive. We aimed to include studies that were explicitly focused at valuing safety. This implies that we excluded studies that used risks in valuing a particular outcome, but did not have valuing safety as the main focus of the paper. Moreover, we focused on monetary valuations, which implies that studies considering risks in another way were also excluded. Consequently, our review did not include studies on ‘wage-risk’ trade-offs, value of a statistical life (VSL) or drug safety. However, multiple literature reviews have recently been carried out for both the VSL and the drug safety literature [5, 18, 35, 53], providing insights from different angles into the safety valuation process.

Moreover, the review process could have been strengthened by having a second author reviewing abstracts, or the inclusion of more types of research, such as theses, papers in a language other than English or grey literature. In a similar vein, the chosen databases have their own limitations; as neither database contains all records from their relevant fields. This limitation was partly mitigated by also including studies based on the reference list of initially included studies. Nonetheless, broadening the set of searched databases might have resulted in a few additional papers. We have no reason to expect that this would significantly change our overall findings. Hence, we would argue that the results from this review are useful in providing first insights into safety valuation. As such, they may inspire more methodological research in this important area, as well as application in economic evaluations of healthcare interventions.

Overall, it has become clear that there is little to no standardisation in safety valuation. Regarding which is ‘the best’ methodology to use, this literature review brings to light more questions than it does answers: What definition of safety is the best for its evaluation? Which stated preference method should be used, CV or DCE, and which methodological issues should be considered in study design? Should the individual or the societal view be applied in the context of valuing public goods? Which covariates should be added to gain the most insight into an individual’s WTP? In other words, there still appears to be a long way ahead before consensus can be attained about a standardised methodology for valuating safety. In the meantime, forthcoming safety valuation research can build upon the findings of this review of the literature, and contribute to the development of more standardised methods by addressing questions about definition of safety, choice and design of method, perspective for valuation, and selection of covariates, thoroughly and clearly.

Concluding, there is no ‘golden standard’ for safety valuation—there are many different approaches to research methods, survey design, biases and context in the literature. Moreover, given the amount of unresolved issues, many aspects of valuing safety are not yet fully understood. What this shows is that there is more work to be done on methodologies for the valuation of safety, theoretically and empirically. That way, it may be able to work towards something more closely resembling a ‘golden standard’ for safety valuation, which is especially relevant in the field of health economics and economic evaluations addressing health related issues. Investing in this important area, therefore, appears to be a safe bet.

## Appendix

### Appendix A—exact search strings

Search Strings:

1. valu* AND (safety OR security OR “uncertainty reduction” OR “risk reduction”).
2. valu* AND (safety OR security OR “uncertainty reduction” OR “risk reduction”) AND review.
3. “shadow price” AND (safety OR security OR “uncertainty reduction” OR “risk reduction”).
4. “shadow price” AND (safety OR security OR “uncertainty reduction” OR “risk reduction”) AND review.
5. “willingness to pay” AND (safety OR security OR “uncertainty reduction” OR “risk reduction”).
6. “willingness to pay” AND (safety OR security OR “uncertainty reduction” OR “risk reduction”) AND review.
7. “willingness-to-pay” AND (safety OR security OR “uncertainty reduction” OR “risk reduction”).
8. “willingness-to-pay” AND (safety OR security OR “uncertainty reduction” OR “risk reduction”) AND review.
9. “willingness to accept” AND (safety OR security OR “uncertainty reduction” OR “risk reduction”).
10. “willingness to accept” AND (safety OR security OR “uncertainty reduction” OR “risk reduction”) AND review.
11. “willingness-to-accept” AND (safety OR security OR “uncertainty reduction” OR “risk reduction”).
12. “willingness-to-accept” AND (safety OR security OR “uncertainty reduction” OR “risk reduction”) AND review.
13. “discrete choice experiment” AND (safety OR security OR “uncertainty reduction” OR “risk reduction”).
14. “discrete choice experiment” AND (safety OR security OR “uncertainty reduction” OR “risk reduction”) AND review.
15. “stated preference” AND (safety OR security OR “uncertainty reduction” OR “risk reduction”).
16. “stated preference” AND (safety OR security OR “uncertainty reduction” OR “risk reduction”) AND review.
17. “revealed preference” AND (safety OR security OR “uncertainty reduction” OR “risk reduction”).
18. “revealed preference” AND (safety OR security OR “uncertainty reduction” OR “risk reduction”) AND review.
19. “contingent valuation” AND (safety OR security OR “uncertainty reduction” OR “risk reduction”).
20. “contingent valuation” AND (safety OR security OR “uncertainty reduction” OR “risk reduction”) AND review.
